# Linkage disequilibrium vs. pedigree: Genomic selection prediction accuracy in conifer species

**DOI:** 10.1371/journal.pone.0232201

**Published:** 2020-06-10

**Authors:** Frances R. Thistlethwaite, Omnia Gamal El-Dien, Blaise Ratcliffe, Jaroslav Klápště, Ilga Porth, Charles Chen, Michael U. Stoehr, Pär K. Ingvarsson, Yousry A. El-Kassaby

**Affiliations:** 1 Department of Forest and Conservation Sciences, Faculty of Forestry, The University of British Columbia, Vancouver, British Columbia, Canada; 2 Pharmacognosy Department, Faculty of Pharmacy, Alexandria University, Alexandria, Egypt; 3 Scion (New Zealand Forest Research Institute Ltd.), Whakarewarewa, Rotorua, New Zealand; 4 Département des sciences du bois et de la forêt, Université Laval, Québec, QC, Canada; 5 Department of Biochemistry and Molecular Biology, Oklahoma State University, Stillwater, OK, United States of America; 6 British Columbia Ministry of Forests, Lands and Natural Resource Operations, Victoria, BC, Canada; 7 Linnean Centre for Plant Biology, Department of Plant Biology, Swedish University of Agricultural Sciences, Uppsala, Sweden; Technical University in Zvolen, SLOVAKIA

## Abstract

**Background:**

The presupposition of genomic selection (GS) is that predictive accuracies should be based on population-wide linkage disequilibrium (LD). However, in species with large, highly complex genomes the limitation of marker density may preclude the ability to resolve LD accurately enough for GS. Here we investigate such an effect in two conifer species with ~ 20 Gbp genomes, Douglas-fir (*Pseudotsuga menziesii* Mirb. (Franco)) and Interior spruce (*Picea glauca* (Moench) Voss x *Picea engelmannii* Parry ex Engelm.). Random sampling of markers was performed to obtain SNP sets with totals in the range of 200–50,000, this was replicated 10 times. Ridge Regression Best Linear Unbiased Predictor (RR-BLUP) was deployed as the GS method to test these SNP sets, and 10-fold cross-validation was performed on 1,321 Douglas-fir trees, representing 37 full-sib F_1_ families and on 1,126 Interior spruce trees, representing 25 open-pollinated (half-sib) families. Both trials are located on 3 sites in British Columbia, Canada.

**Results:**

As marker number increased, so did GS predictive accuracy for both conifer species. However, a plateau in the gain of accuracy became apparent around 10,000–15,000 markers for both Douglas-fir and Interior spruce. Despite random marker selection, little variation in predictive accuracy was observed across replications. On average, Douglas-fir prediction accuracies were higher than those of Interior spruce, reflecting the difference between full- and half-sib families for Douglas-fir and Interior spruce populations, respectively, as well as their respective effective population size.

**Conclusions:**

Although possibly advantageous within an advanced breeding population, reducing marker density cannot be recommended for carrying out GS in conifers. Significant LD between markers and putative causal variants was not detected using 50,000 SNPS, and GS was enabled only through the tracking of relatedness in the populations studied. Dramatically increasing marker density would enable said markers to better track LD with causal variants in these large, genetically diverse genomes; as well as providing a model that could be used across populations, breeding programs, and traits.

## Introduction

With genotyping costs at the lowest they have ever been (and still on a decreasing trajectory), genomic selection (GS) becomes an ever-more viable option for forest tree breeders. This should subsequently result in beneficial gains to the industry in terms of improved wood quality, yield per unit time (generational turnover), and stress tolerance (biotic and abiotic) [1,2]. In a deviation from marker-assisted selection (MAS) [3], rather than attempting to identify significant trait-loci relationships, GS employs all available marker information simultaneously to predict traits performance [4]. GS experimental results have been promising to date, with prediction accuracies higher than those of MAS [5].

One of the major determinants of GS success is the relationship between effective population size (*N*_*e*_) and marker density [6]. Falconer and Mackay [7] succinctly describe *N*_*e*_ as the number of randomly mating individuals that would cause the observed inbreeding rate of a population. As a result of inbreeding, which is more likely at low *N*_*e*_, allele frequencies become skewed. In this situation (at low *N*_*e*_) certain individuals have an increased chance to reproduce, causing genetic drift as their genes are passed more frequently onto the next generation [6]. Over time and over multiple generations of these conditions those alleles may become fixed, as genetic diversity decreases [7]. Low *N*_*e*_ populations are subject to stronger drift, which in turn is one of the major driving forces influencing linkage disequilibrium (LD) between loci. Lower *N*_*e*_ populations have higher LD, and this LD between markers and trait QTLs is essential for the prediction of trait performance from markers [6]. The success of GS is highly dependent on the marker-QTL LD and this is largely determined by the extent to which the training and validation populations are related. A certain amount of caution is also required during the implementation of GS as LD will decay subsequent to every round of breeding, due to recombination, although this can be overcome by employing dense marker arrays. It should be emphasized that GS may require alternative delivery methods for tree improvement, as the current seed orchard production pathway and its sexual-production mode effectively breaks down the marker-QTL LD. Lin et al. [6] point out that *N*_*e*_ can be artificially reduced by using half-sib or within-family populations. This is a good technique to use on outbred species, such as forest trees, when applying GS.

The determination of the number of markers required for GS is largely based on the occurrence of LD, which in turn is determined by population structure and *N*_*e*_. Solberg et al. [8] did some initial investigations into marker density effect, comparing two types of marker: SSR-like multiallelic markers versus SNP-like biallelic markers. They found a general increase in prediction accuracy as marker density increased in relation to *N*_*e*_, however they failed to reach a plateau with the numbers available (2*N*_*e*_ SSR markers per Morgan or 8*N*_*e*_ SNP markers per Morgan). Meuwissen [9] proposed that a minimum of 10*N*_*e*_*L* markers should be used to obtain accurate predictions in GS, where *L* is the total length of the genome in Morgans. As Lin et al. [6] discuss, this should mean that for a population of outbreeding apple (*Malus* sp.) trees with an assumed *N*_*e*_ of 1,000 and a genome of approximately 13 Morgans, 130,000 markers are necessary for accurate GS predictions. However Kumar et al. [10] successfully carried out GS in apple variety *Malus domestica* Borkh with an accuracy of 0.7, using only 2,500 markers. This can be attributed to the bi-parental design used [9]. Within-family designs call for fewer markers since larger (but fewer) chromosomal segments are shared by family members, and it is these that need to be tracked [11]. Grattapaglia and Resende [12], using a deterministic approach found that for GS to be effective, a marker density of ~2 markers/cM is required when *N*_*e*_ is no greater than 30 and larger *N*_*e*_ may require up to 20 markers/cM. Yet higher marker densities may be required for situations in which the training and validation populations are not derived genetically from the same base population [9]. Using Grattapaglia and Resende's [12] calculations, and an assumed map length of 2,000 cM [13–16], we should aim to use 4,000 markers for investigations into Douglas-fir (*Pseudotsuga menziesii* Mirb. (Franco)) with an *N*_*e*_ ~21. By the same reasoning we should aim to use up to 40,000 markers for an Interior spruce (*Picea glauca* (Moench) Voss x *Picea engelmanni*i Parry ex Engelm.) population, with an *N*_*e*_ ~93. Indeed Howe et al. [17] concluded that a density of 2.5 markers per cM (5,000 SNPs/2,000 cM), should provide effective GS results in populations no larger than *N*_*e*_ ~30. These numbers, for populations with low *N*_*e*_, are in line with Meuwissen's [9] determination that 10N_e_*L* markers should be used as a minimum, which gives us 10×21×20 = 4,200 markers for Douglas-fir. Yet using the same calculation from Meuwissen [9] for Interior spruce, gives us a recommended minimum of 18,600 markers, less than half of that recommended by Grattapaglia and Resende's [12] calculation.

Ma et al. [18] investigated the effect of marker selection on prediction accuracy of GS in soybean (*Glycine max L*.). They tested three methods of marker selection: random sampling, haplotype block analysis, and evenly sampling markers. They found that for plant height, only marginal differences in prediction accuracy were obtained with the three sampling methods. However, for grain yield, the haplotype block analysis out-performed the other two methods by about 4%. This preselection method offers a comprehensive, yet cost-efficient, option for implementing GS by reducing the number of SNPs required to just one SNP per haplotype block plus those not contained within blocks. However, an in-depth understanding of the structure and LD across the genome is required for this. For this reason, we have concentrated only on the random sampling method for Douglas-fir and Interior spruce where current genome assemblies are highly fragmented and not conducive to analyses of genome wide patterns of LD.

**Fig 1:**
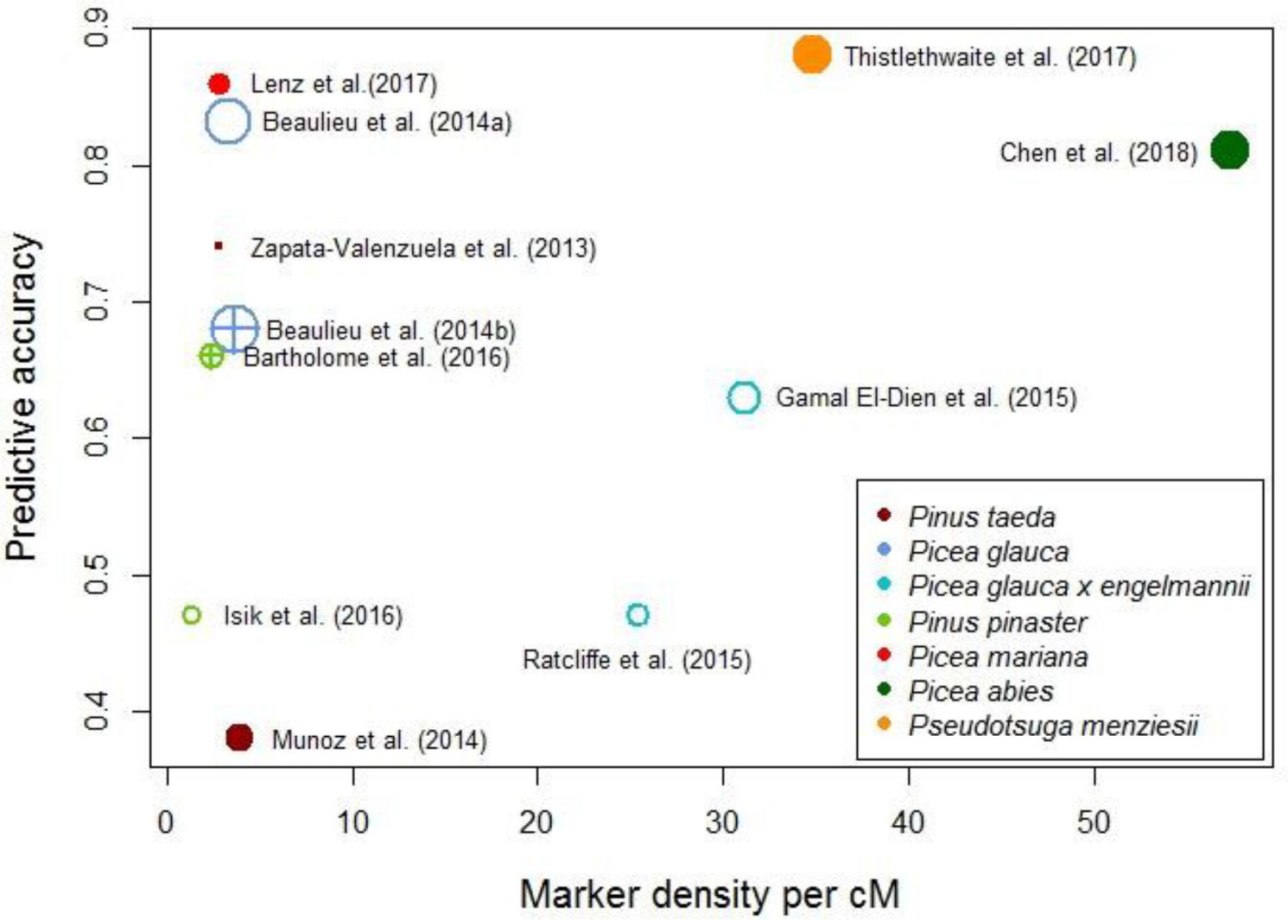
Scatterplot of metadata concerning height prediction accuracy, from various sources of GS studies [19,21–30] in forestry of conifer species. Filled points represent studies using full-sib populations; empty points represent studies using half-sib populations; empty points with crosses represent studies using full and half-sib populations. Point diameter is a function of sample size. Marker density was calculated based on genetic map lengths estimated in the following studies: *Pinus taeda* [31], *Picea glauca* [32], *Picea glauca* x *engelmannii* (based on white spruce data) [32–34], *Pinus pinaster* [35], *Picea mariana* [36], *Picea abies* [37].

The two species studied here are representative of full-sib (Douglas-fir) and half-sib (Interior spruce) populations. Their differing pedigree structure should be reflected in the prediction accuracies we obtain through GS. Previously, using full-sib families, GS prediction accuracy has been shown to be moderate to high in general [19,20], and in Douglas-fir specifically [21]. This is considered to be a result, primarily, due to long range LD arising from the increased levels of relatedness within families. Short range LD is not considered a strong influence in these circumstances. By comparison, using half-sib families has previously resulted in low to moderate GS prediction accuracies in general [22], and in Interior spruce specifically [23,24]. Higher *N*_e_ and subsequently lower LD are thought to impede prediction accuracies in studies based on these half-sib populations. Larger *N*_*e*_ leads to more recombination and therefore more diversity within a population. Drift associated with small population size is not a significant factor under these conditions (open-pollinated and highly outcrossing species) and subsequently do not significantly contribute to the build-up of LD. Nonetheless, as can be seen in Fig 1, high GS prediction accuracies are not exclusive to full-sib studies and *vice versa*.

It is, however, becoming seemingly more apparent that perhaps LD is not the main driving force in all GS studies [21,30]. Studies concerning those species with larger genomes may find that relatedness rather than LD is the most important factor influencing prediction accuracies. As de los Campos et al. [31] describe, the success of GS relies upon the similarity of the realised genomic relationships at the marker and QTL levels. The number of independently segregating segments determines the coefficient of variation of these relationships across the genome. In unrelated individuals, this is a product of population-wide LD whereas between family members, this is a product of within family disequilibrium. Here we investigate the effect of marker density on GS prediction accuracy in two conifer species (Douglas-fir and Interior spruce) with differing pedigree structures (full- and half-sib family structure). The main underlying assumption of GS studies is the presence of LD between markers and causal genes, thus the derived predictive models are transferable and can be used for phenotypic prediction of genotypes but non-phenotyped individuals. However, if the obtained predictive accuracy is the result of increased pedigree resolution, then these models should be used with caution, as their predictive accuracy is pedigree dependent.

## Results

### Marker number effect

**Fig 2:**
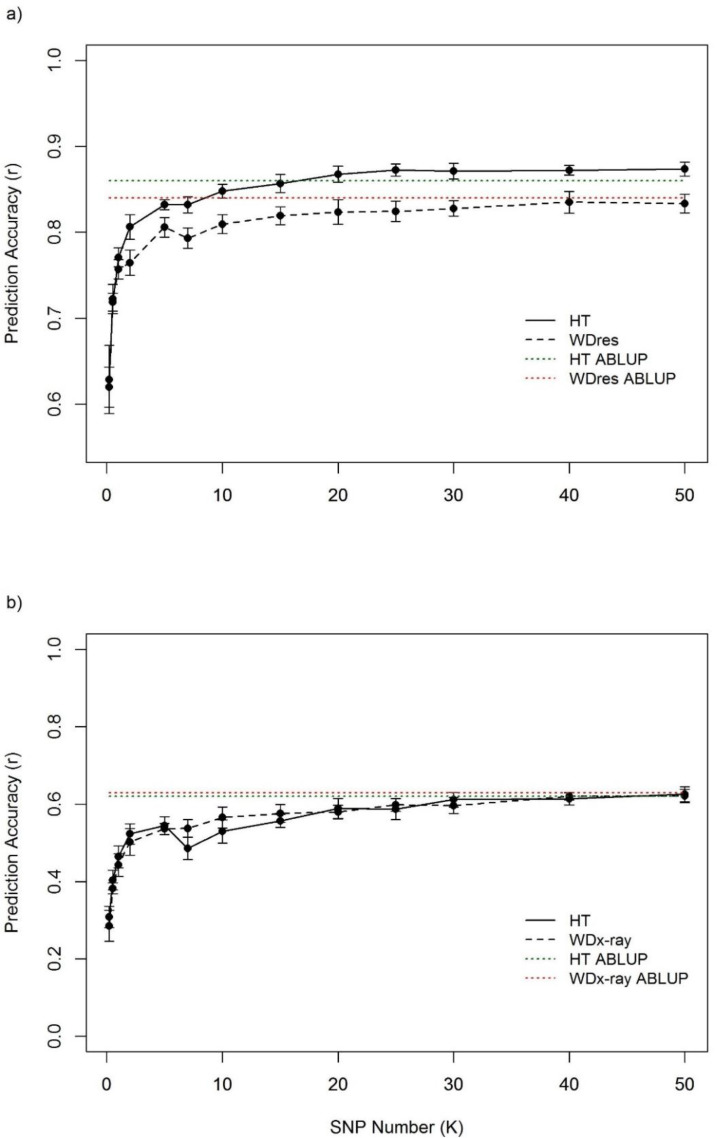
Effect of marker density on RR-BLUP prediction accuracy for multi-site data for height and wood resistance, with ABLUP for comparison, for: a) Douglas-fir (HT at 35 years, WD_res_ at 38 years), and b) Interior spruce (HT at 38 years, WD_X-ray_ at 38 years).

For Douglas-fir, the average prediction accuracy for height genomic estimated breeding values (GEBVs) ranged from 0.63 (standard error: ±0.040) to 0.87 (±0.008), and for Interior spruce 0.31 (±0.028) to 0.63 (±0.019), representative of GS testing SNP sets of 200 and 50,000 SNP number totals, respectively, for each species. Similarly, for Douglas-fir, the average accuracy of wood density GEBVs ranged from 0.62 (±0.023) to 0.83 (±0.011), and for Interior spruce, 0.29 (±0.040) to 0.62 (±0.017), using SNP set totals of 200 and 50,000, respectively. Accordingly, the effect of marker number on the prediction accuracy of multi-site cross-validation data, showed a clear trend of increased predictive accuracy with increasing marker density. However, the magnitude of prediction accuracy gains starts to plateau around a threshold of around 10,000–15,000 markers for both Douglas-fir and Interior spruce (Fig 2). Despite the random selection of markers, there is also little variation in predictive accuracy among SNP sets of the same SNP number total, as indicated by the small error bars in Fig 2. On average, the accuracies for height increased by a factor of 1.03 and 1.08, respectively, when the number of markers was doubled, for Douglas-fir and Interior spruce, respectively. For wood density the accuracies increased on average by a factor of 1.02 and 1.07, respectively, when the number of markers was doubled, for Douglas-fir and Interior spruce, respectively. Fig 2 provides a graphical summary of the results, depicting the average predictive accuracy of 100 replicates per SNP set and 10 random replicates for each SNP set total, as well as their standard errors.

### Pedigree and relatedness effect

The full-sib structure related to the Douglas-fir models outperformed those of the half-sib structure of the Interior spruce models for all SNP set totals (Fig 2). The prediction accuracies for the full-sib models (Douglas-fir) were for height and wood density, respectively, on average 1.58 and 1.54 times larger than for the half-sib models (Interior spruce). Prediction accuracy varied more within traits when using half-sibs compared to full-sib pedigrees, as represented by the SNP set total error bars in Fig 2. On average, the standard deviations for half-sib models were 2.59 times larger than those of the full-sib models for height, and 1.75 times larger for wood density.

## Discussion

Generally, prediction accuracies for height and wood density GEBVs of both Douglas-fir and Interior spruce, increased with increasing number of SNPs (Fig 2). The data suggests that increasing the number of markers will therefore provide more accurate predictions. However, in light of current genotyping costs and efficiency, it is prudent to use as few markers as possible without loss of significant accuracy. Ideally, this would mean that the minimum number of markers should be the same as the number of independent linkage blocks. This number may vary according to the type of breeding population, and type of genomic data collected. Although the saturation point for our data was around 10,000 markers for both Douglas-fir and Interior spruce using EBVs as the model input, certainly the *N*_*e*_ of the populations need to be considered. Data with a higher *N*_*e*_ (>93) may require more markers to obtain similar prediction accuracies.

The observed GEBV coalesce similar results obtained in previous breeding program studies and generalized simulations [8,22,30,38–42]. The GS prediction accuracies for both height and wood density, parallel those obtained by ABLUP and by GS in previous studies on these species [21,23]. These results from models trained on EBVs led us to conclude that, even at a relatively low density, the markers used were able to capture the genetic relationship as effectively as the pedigree, and in the case of HT at 35 years for Douglas-fir, more effectively. As a result of this finding, future selections may be conducted more efficiently and without the need for a structured pedigree, thereby speeding up the breeding process by eliminating the need to conduct specific crosses [43]. Furthermore, prior studies in Douglas-fir modeling de-regressed EBVs, where parental averages are removed, showed extremely low GS prediction accuracies and largely undetectable LD [21,44].

The role of short-range marker-QTL LD on GS prediction accuracy was imperceptible compared to the effect of relatedness. This is a somewhat anticipated result given that most conifers are relatively undomesticated even within breeding programs, and are known to be highly outbred species with large *N*_*e*_. These characteristics actively preclude the buildup of population-wide LD [45,46]. Corroborating evidence for this can been seen in Fig 2, where the variance of the prediction accuracies is modest despite SNPs being randomly selected. This leads us to believe that the SNPs are tracking genetic relatedness as opposed to marker-QTL LD. In addition, other studies have drawn similar conclusions that marker selection strategies have little to no effect on prediction accuracy for growth and wood quality traits [19,30,40]. That is to say, that marker sets made up of only those markers with the largest effects show only a minor advantage over using all markers available [19,30]. As reported by Lenz et al. [30], this observation is likely due to those high-effect markers having higher mean allele frequecies, and hence higher information value allowing them to trace genetic relationships efficiently. In addition, Zapata-Valenzuela et al. [40] found no discernable difference in prediction accuracy when using subsets of markers rather than the whole compliment of markers available to them. This was regardless of whether or not those markers were in association with the trait in question. Going further, considering the metadata presented in Fig 1, marker density at the levels currently applied appears to have little to no bearing on GS prediction accuracy. Studies with less dense marker coverage, in some cases over 20 times less dense than the highest, display similarly high prediction accuracies. Since it is unlikely that such few markers could account for LD with most causal variants across such vast genomes, it is an indication that these markers are tracking something else than LD between markers and QTLs (i.e., pedigree).

Even in species, which do not display the complexity of conifer genomes, similar trends due to marker density reduction have been seen. Using two Eucalyptus species and their F_1_ hybrids, Tan et al. [41] found no major advantage in using more than 5,000 SNPs compared to using the full data set of 41,304 SNPs available to them. Lorenz et al. [47] noted that in a barley (*Hordeum vulgare* L.) population with high LD, the number of markers used could be reduced to 384, with minor impact on prediction accuracy for disease resistance (note, barley is a selfer). Marker selection via a *k*-means clustering algorithm to sample LD space had only very modest advantages over random sampling. Additionally, in dairy breeding, multiple studies have shown that prediction accuracy can be maintained despite significantly reducing marker density [48,49]. In these cases, it is the effect of low *N*_*e*_ [11] which is driving the prediction accuracy. With low *N*_*e*_, fewer independent genomic segments arise, decreasing the number of markers required to track these segments [42]. These populations all exhibit closed breeding and subsequently have lower *N*_*e*_ than our sampled populations. Lowering *N*_*e*_ in this way would be disadvantageous when predicting across families.

Complex traits are thought to be largely governed by noncoding variants seemingly affecting gene regulation and expression [50,51]. The number of such variants are thought to be very large, evenly distributed across the genome and have small effect sizes. The heritability of complex traits is thus similarly spread out throughout the entire genome [50]. The upshot of this is the implication that a large proportion of all genes contribute to variation in complex traits. This is at odds with the expectation that trait variants are located within specific and biologically relevant genes and pathways [50]. Yet Tan et al. [41] generally found that SNPs located in intergenic regions provided slightly better prediction accuracies over those located within/near genic regions, or when using all SNPs available. They attributed this to a slower decline of LD in intergenic regions compared to other genome locations, allowing markers in intergenic regions to effectively trace QTLs over longer genomic segments than markers in genome regions with higher rates of LD decay. Similarly Boyle et al. [50], in their summary report, state that SNPs that contribute most to the heritability are often spread widely across the genome and are not closely located to genes with trait-specific functions.

Although genome-wide variation in LD is virtually unknown in conifers, data from other plant species suggest that LD is higher but also more variable in intergenic compared to genic regions. There are several reasons for this. First, intergenic regions in many species are replete with repetitive elements, mainly various Long Terminal Repeats (LTR) transposable elements [52,53], and such regions are often highly heterochromatic and show reduced cross-over rates. Second, complex structural variation generated by the action of repetitive elements will further limit cross over due to lack of sequence homology in intergenic regions, directing recombination to genomic regions with high gene densities. Both features are well documented in maize, a species that also has a relatively high repetitive genome fraction [52,54]. As noted above, levels of linkage disequilibrium in conifers are poorly studied to date. Small, targeted re-sequencing of exomic regions have found that LD decays relatively rapidly, over a few kilobases at the most [53,55,56]. This is to be expected if most recombination is largely directed towards genic regions. However, since genic regions only constitute at most a percent or so of a typical conifer genome, these rates are likely not representative for most of the genome. As an example, Fu et al. [52] concluded that the repetitive DNA in maize, while constituting the bulk of the genome, likely contributes little if anything to genetic length. Given these observations, where current genome assemblies are highly fragmented and not conducive to analyses of genome wide patterns of LD, it is perhaps not unusual that, despite relatively large marker totals in use here (and an even greater total used in Thistlethwaite et al. [21]) and for Douglas-fir those markers being located in the exonic regions, LD was not successfully traceable.

To further elucidate the role of markers on resolving the pedigree which in turn affected height and wood density predictive accuracies for the two studied species. It should be noted that the Douglas-fir full-sib outperformed the Interior spruce half-sib structure (on average full-sibs were 1.58 and 1.54 times larger than half-sib for height and wood density, respectively) (Fig 2). These results are indicative of the markers ability in resolving higher relationships among the 37 full-sib families (within and cross families due to common parentageas well as hidden inbreeding). On the other hand, while the Interior spruce has fewer families (25 half-sibs), their open-pollinated nature precluded the development of finer relationships as each seed-donor originated from different location, thus distant relationships were not present. This is also clearly demonstrated by the differences in full- and half-sib families *N*_e_ values (21 vs. 93 for the Douglas-fir and Interior spruce, respectively).

## Conclusions

Although advantageous within a population, reducing marker density may not be the most effective or economical method of carrying out GS, especially in conifers. As mentioned previously we have yet to trace LD with the current array of markers available, only genetic relationships, which is not the intended use of GS. Given the genetic diversity of conifer species, it would perhaps be more prudent to create denser marker arrays that can be used across populations and breeding programs [57]. Increasing the number of markers in such a way could enable us to tease apart the impact of genetic relationship from LD and to investigate multiple traits including unanticipated traits [22]. If this can be achieved, the higher density of markers would offset somewhat the effects of marker-QTL LD decay due to selection and recombination over multiple generations [8,22,57]. Therefore, low density marker arrays would have more impact in more advanced breeding programs [57].

## Material and methods

### Experimental populations

Predictive models for GS were trained on two progeny testing populations. The first population consist of 38-year-old coastal Douglas-fir (*Pseudotsuga menziesii* Mirb. (Franco)). This population was originally established by the Ministry of Forests, Lands and Natural Resource Operations of British Columbia, Canada in 1975 and it is made up of 165 full-sib families (54 parents), from which 37 families were selected for sampling from three test sites (**Adams** (Lat. 50 24’42” N, Long. 126 09’ 37”W, Elev. 576 mas), **Fleet River** (Lat. 48 39’25” N, Long. 128 05’05” W, Elev. 561 mas), and **Lost Creek** (Lat. 49 22’15” N, Long. 122 14’07” W, Elev. 424 mas)) giving a total of 1,372 trees (N≈500 per site).

The second population consisted of 1,126 38-year-old Interior spruce trees (*Picea glauca* (Moench) Voss x *Picea engelmanni*i Parry ex Engelm.) (N≈375 per site). This progeny test trial was established in 1972/73 by the Ministry of Forests, Lands and Natural Resource Operations of British Columbia Canada and is made up of 181 open-pollinated families, of which the best performing 25 families were selected based on their growth attributes. The trial is located on three sites (**Aleza Lake** (Lat. 54° 03′ 15.7″ N, Long. 122° 06′ 35.4″ W, Elev. 700 mas), **Prince George Tree Improvement Station (PGTIS**) (Lat. 53° 46′ 17.9″ N, Long. 122° 43′ 07.6″W, Elev. 610 mas), and **Quesnel** (Lat. 52° 59′ 27.2″ N, Long. 122° 12′ 30.6″ W, Elev. 915 mas)).

Access to Douglas-fir and Interior spruce progeny test trials was granted by The Ministry of Forests, Lands and Natural Resource Operations of British Columbia, Canada, and all ethics standards have been met.

### Phenotyping and genotyping

Mid-rotation height measurements of the sampled trees were recorded: at age 35 for the Douglas-fir population, and at age 38 Interior spruce (HT: in meters). Estimated breeding values (EBVs) for HT were obtained in ASReml 4.0 [58] and used as phenotypes for the genomic prediction analysis. Wood density (WDres) measurements for the Douglas-fir population were taken indirectly, using the average of resistance measurements obtained with a Resistograph^®^ (Instrumenta Mechanik Labor, Germany). Recordings from the Resistograph^®^ were scaled by DBH measurements to obtain wood density indices following El-Kassaby et al. [59]. Wood density in the Interior Spruce population was measured directly in kg/m^3^ using X-ray densitometry (WD_X-ray_), which uses increment cores extracted from the trees.

Genotyping of the Douglas-fir samples, using whole exome capture, was performed in a commercial facility (RAPiD Genomics^©^, Florida, US), probes were designed based on the available Douglas-fir transcriptome assembly [17]. A total ‘pool’ of 56,454 SNPs, with <0.40 heterozygosity, was used in this study. For a complete discription of the genotyping process see Thistlethwaite et al. [21] and Neves et al. [60] for the exome capture methodology respectively.

The Interior spruce samples were genotyped via multiplexed, high-throughput Genotyping-by-Sequencing (GBS) following Elshire et al. [61] and Chen et al. [62], on the Illumina HiSeq 2000 at the Cornell University Genomics Core Laboratory (Gamal El-Dien et al. [23]).

### Effective population size estimation

The effective population size (*N*_e_) for the Douglas-fir and spruce were estimated using an Excel program developed by Dr. M. Lstiburek (Faculty of Forestry and Wood Sciences, Czech University of Life Sciences Prague, Prague, Czech Republic) that was based on Lindgren et al. [63] status number concept.

### Random marker sampling

The effect of the number of markers on predictive accuracy was ascertained by carrying out a random sampling method for choosing markers from the total ‘pool’ containing 56,454 SNPs for Douglas-fir and 62,190 for Interior spruce. Sets with SNP totals in the range of 200–50,000 were tested and replicated 10 times, randomly sampling SNPs for each repetition. The cross-validation processes of the RR-BLUP model was then performed using these randomly sampled SNP sets. This analysis was carried out on the height and wood density phenotypes. Assuming a genome length of ~2,000cM for both Douglas-fir [13] and Interior spruce [14–16] (an approximation based on *Picea glauca*, *Pinaceae* data), the average marker densities tested ranged from 0.05–25 markers/cM.

### Estimated Breeding Value (EBV) calculation and ABLUP accuracy

EBVs were calculated in ASReml 4.0 [58] via linear mixed model analysis. For the Douglas-fir population the following model was used:

$$y=X\beta +Z_{1}a+Z_{2}sa+Z_{3}s(rep)+Z_{4}sf+Z_{5}f+e$$ (1)

Where *y* is the phenotypic trait measurement, *β* is a vector of fixed effects (including mean and site effects), *a* is a vector of individual random additive genetic effects ~ N(0, ***A***σ_a_^2^), *sa* is a site x additive genetic interaction ~ N(0, ***I***σ_sa_^2^), *s(rep)* is a vector of the block effect within site ~ N(0, ***I***σ_s(rep)_^2^), *sf* is a random effect site x family interaction ~ N(0, ***I***σ_sf_^2^), *f* is the effect of family ~ N(0, ***I***σ_f_^2^), and *e* is the random residual effect ~ N(0, ***I***σ_e_^2^), and ***X***, ***Z***_**1-5**_ are incidence matrices assigning fixed and random effects to each observation. ***I*** is the identity matrix and ***A*** the average numerator relationship matrix.

Narrow-sense heritability was calculated as h^2^ = σ_a_^2^ / (σ_a_^2^ + σ_sa_^2^ +σ_sf_^2^ + σ_f_^2^ + σ_e_^2^), where σ_a_^2^, σ_sa_^2^, σ_sf_^2^, σ_f_^2^ and σ_e_^2^ are the variances of additive genetic, site x additive genetic, site x family, family, and residual effects, respectively.

For the Interior Spruce population, a similar mixed model was used, without family effect:

$$y=X\beta +Z_{1}a+Z_{2}sa+Z_{3}s(rep)+e$$ (2)

where *y* is the phenotypic measurement of the analyzed trait, *β* is a vector of fixed effect (i.e., the overall mean and the site effect), *a* is a vector of random additive effects ~ N(0, ***A***σ^2^_a_), *sa* is a site x additive genetic interaction ~ N(0, ***I***σ^2^_sa_), *s(rep)* is a vector of the block effect within site ~ N(0, ***I***σ_s(rep)_^2^), *e* is a the random residual effect ~ N(0, ***I***σ^2^_e_), and ***X***, ***Z***_**1-3**_ are incidence matrices assigning fixed and random effects to each observation. ***I*** is the identity matrix and ***A*** the average numerator relationship matrix.

Narrow-sense heritability was calculated as *h*^2^ = σ_a_^2^/ (σ_a_^2^ + σ_sa_^2^ + σ_e_^2^), where σ_a_^2^, σ_sa_^2^, and σ_e_^2^ are the variances of additive genetic, site x additive genetic, and residual effects, respectively.

ABLUP cross-validation for both species was performed in ASReml R v4.0 [58]. Ten fold cross-validation was performed, using randomly sampled individuals from all 3 sites (Adams, Fleet River and Lost Creek for Douglas-fir; Aleza Lake, PGTIS and Quesnel for Interior spruce) to construct the model, and the remainder to compose the validation set. Prediction accuracy for ABLUP was calculated as the correlation between the EBVs from the validation sets and their original EBVs calculated from Eqs 1 and 2, for Douglas-fir and Interior spruce respectively. ABLUP prediction accuracy was compared to GS prediction accuracy for all SNP set totals.

### Genomic selection and cross-validation

The GS method used in the analysis was ridge regression (RR-BLUP) [64], and was implemented using the R package ‘bigRR’ [65]. The genomic predicted EBVs (GEBVs) for height, were calculated as the sum of all marker effects within each individual tree. Marker effects were estimated using the following mixed model, from Henderson [66]:

$$y_{EBV}=1\mu +Zg+e$$ (3)

where ***y_EBV_*** is the vector of *n* tree EBV records for height, **1** is a vector of 1, ***μ*** is the intercept, ***g*** is the vector of random marker effects, ***Z*** is the design matrix for the random marker effects, and ***e*** is the residual random effects vector. In the RR-BLUP procedure, the residuals and marker effects are presumed to follow normal distributions with constant variance, i.e. *e* ~ $N(0,I\sigma_{e}^{2})$ and *g* ~ $N(0,I\sigma_{g}^{2})$, where ***I*** is an identity matrix. Marker effect solutions are calculated according to the following equation:

$$\hat{g}={\left(Z^{\prime }Z+ʎI\right)}^{-1}Z\prime y$$ (4)

where *ʎ* = $\sigma_{e}^{2}/\sigma_{g}^{2}$ is the ridge penalization parameter. Marker effects are assumed to be distributed equally, and as such all are uniformly shrunk towards zero.

Predictive accuracy was used to estimate the performance of this GS method. Predictive accuracy was determined as the mean of the replications of the Pearson product-moment correlation between estimated breeding values (EBVs) of the validation set and their genomic estimated breeding values (GEBVs) or r(EBV,GEBV). Validation was applied as a replicated randomly assigned 10-fold cross validation repeated 10 times in which 9/10 folds were used to train the model, the other fold constituting the validation population. Information from the 3 sites were pooled.
